# Association of 17q24 rs1859962 gene polymorphism with prostate cancer risk: A systematic review and meta-analysis

**DOI:** 10.1097/MD.0000000000018398

**Published:** 2020-01-17

**Authors:** Feiqiang Ren, Peihai Zhang, Ziyang Ma, Ling Zhang, Guangsen Li, Xiaopeng Huang, Degui Chang, Xujun Yu

**Affiliations:** a Chengdu University of Traditional Chinese Medicine; b The Urology and Andrology Department, Hospital of Chengdu University of Traditional Chinese Medicine; c The Andrology Department, The School of Medical and Life Sciences, Chengdu University of Traditional Chinese Medicine, Chengdu, Sichuan Province, P. R. China.

**Keywords:** 17q24 rs1859962 polymorphism, prostate cancer, single-nucleotide polymorphism

## Abstract

**Background:**

Recently, several genome-wide association studies have demonstrated a cumulative association of 17q24 rs1859962 gene variants with prostate cancer (PCa) risk, but conflicting results on this issue have been reported. Hence, we performed a systematic literature review and meta-analysis to assess the association between 17q24 rs1859962 gene and PCa risk.

**Methods:**

Systematic literature searches were conducted with PubMed, EMBASE, Science Direct/Elsevier, CNKI, and the Cochrane Library up to January 2019 for studies focusing on the association of 17q24 rs1859962 gene polymorphism with PCa risk. Meta-analysis was performed with Review Manager and stata software. Combined OR were identified with 95% confidence intervals (95% CI) in a random or fixed effects model.

**Results:**

Eight studies were identified, including 7863 cases of PCa patients and 17122 normal controls. Our results revealed significant associations between the 17q24 rs1859962 gene polymorphism and PCa in all genetic models (*P* < 0.05). The combined odds ratios and 95% confidence intervals were as follows: Additive model (odds ratios [ORs] 1.44, 95%, confidence interval [CI] [1.32, 1.57]); Codominant model (ORs 1.22, 95% CI [1.08, 1.39]); Dominant model (ORs 1.25, 95%, CI [1.17, 1.34]); recessive model (ORs 1.27, 95% CI [1.18, 1.36]); allele model (ORs 1.32, 95% CI [1.12, 1.55]).

**Conclusion:**

The present study supports the proposed association between the 17q24 gene rs1859962 and PCa progression. Specifically, this polymorphism is suggested to be a risk factor of PCa. However, studies with larger sample sizes are needed to better illuminate the correlation between 17q24 rs1859962 gene polymorphism and PCa.

## 1 Introduction

Prostate cancer (PCa) is one of the most common cancers affecting men worldwide, and causes more than 250,000 deaths annually.^[1,2]^ Although PCa is the most common noncutaneous tumor in developed countries, its etiology remains poorly understood.^[3,4]^ Identifying risk factors for PCa is critical for developing interventions and improving our understanding of the biology of this disease.

Risk factors for PCa increase with age, ethnic background, and familial history of PCa.^[5]^ A genome-wide association study (GWAS) found at least 35 loci related to PCa.^[6,7]^ Testing of these risk alleles across populations is important.^[8]^ Approximately 42% of the risk of PCa can be explained by heritable genetic factors.

Chromosome 17q24 belongs to the non-coding pathogenic gene located in the long arm region 2 and 4 of chromosome 17 with a total length of about 600 kb Genes, its biological expression mechanism is not yet clear. SOX9 (SRY (sex determining region Y)-box 9), which is located in relatively close proximity (1 Mb) to the 17q24 rs1859962 risk variant. It is an important gene associated with early embryonic development, it does not directly regulate testicular development, but occurs through the combined action of the SRY gene on the male Y chromosome.

The long arm of chromosome 17 has been reported in several linkage studies of PCa,^[9–14]^ several large-scale GWASs have reported these SNP variants and PCa risk correlation, but conflicting results on this issue have been reported. Some studies reported that such a polymorphism may increase the risk of PCa, but others did not.^[15–22]^ Therefore, we systematically reviewed the available literature and performed a meta-analysis to evaluate the association of the 17q24 rs1859962 gene polymorphism with PCa risk, which might provide valuable insights on the biology of PCa.

## 2 Materials and methods

### 2.1 Literature search

This meta-analysis was restricted to published studies that investigated the association between the 17q24 rs1859962 gene polymorphism and PCa risk. Two independent reviewers searched PubMed, EMBASE, Science Direct/Elsevier, MEDLINE CNKI, and the Cochrane Library from their inception until January 2019; no restrictions were placed on the language of the report or the study type. The search terms combined text words and MeSH terms. For example, the search terms for the 17q24 rs1859962 gene were “17q24 rs1859962 gene,” “chromosome 17,” “17q24 gene,” “rs1859962,” or “17q gene,” “17q rs1859962 gene,” those for prostate cancer were “prostate cancer,“ “prostatic neoplasms,” “cancer of prostate,” “cancer of the prostate,” “neoplasms, prostate,” “neoplasms, prostatic,” “prostate neoplasms,” “prostatic cancer,” or “PCa”; and those for the polymorphism were “SNP,” “single-nucleotide polymorphism,” “polymorphism,” “variation,” or “mutation.” All related articles and abstracts were retrieved. In addition, references cited within relevant reviews were retrieved manually; the search only focused on full articles.

### 2.2 Eligibility criteria

Inclusion criteria: Studies were included if they tested the association of 17q24 rs1859962 gene variants with PCa. The case groups were PCa patients, while the controls were male, no family history of PCa, negative digital rectal examination, and PSA level <4 ng/mL. Genotyping for the 17q24 rs1859962 gene SNP was conducted using polymerase chain reaction-restriction fragment length polymorphism. Available data were extracted from the article, including eligible and genotyped cases and controls, and the numbers of cases and controls for each 17q24 rs1859962 genotype.

Exclusion criteria: Studies were excluded if they involved case reports; were only published as abstracts, reports from meetings, or review articles; lacked a control population; lacked data on genotype frequencies; or duplicated previous publications.

### 2.3 Study selection and validity assessment

Two independent reviewers screened the titles and abstracts of all citations obtained from the literature search. All relevant studies that appeared to meet the eligibility criteria were retrieved. If an ambiguous decision was made based on the title and abstract, the full text was analyzed. The final decision regarding the eligibility of studies was made by reviewing the articles. Disagreements were resolved by consensus or a third reviewer. Two reviewers completed the quality assessment according to the primary criteria for nonrandomized and observational studies of the Newcastle–Ottawa quality assessment scale in meta-analyses.

### 2.4 Data extraction and statistical analysis

The following data were extracted from the papers by 3 reviewers: authors, year of publication, country, number, and genotyping methods, outcomes of eligible and genotyped cases and controls, and the numbers of cases and controls for each 17q24 rs1859962 genotype. Disagreements were resolved by consensus. Quantitative meta-analysis was performed by 2 reviewers using Review Manager (RevMan) software (version 5.2; The Nordic Cochrane Centre, The Cochrane Collaboration, 2012, Copenhagen, Denmark) and Stata software (version 12.0; College Station, TX). Available data were analyzed in the meta-analysis.

The combined odds ratio (OR) and its 95% confidence interval (CI) were calculated. Heterogeneity was assessed using the *P*-value and the *I*-square statistic (*I*^2^) in the pooled analyses, which represents the percentage of total variation across studies. If the *P*-value was less than .1 or the *I*^2^-value was greater than 50%, the summary estimate was analyzed in a random-effects model. Otherwise, a fixed-effects model was applied. To reduce I error probability, using 5 genetic models for each genotype repeat the comparison several times in pairs. Allele model was calculated for wild type homozygotes versus heterozygotes and mutant type homozygotes, wild type homozygotes versus mutant type homozygotes, wild type homozygotes and heterozygote versus mutant type homozygote. Additive mode was calculated for wild type homozygotes versus mutant type homozygotes. Dominant model was calculated for mutant type homozygote versus wild type homozygotes and heterozygote. Recessive model was calculated for heterozygotes and mutant type homozygotes versus wild type homozygotes. Codominant model was calculated for mutant type versus mutant type homozygotes. The association between 17q24 rs1859962 polymorphism in the 17q24 rs1859962 gene and PCa risk was investigated in an allelic model (T vs G), additive model (TT vs GG), dominant model (TT and TG vs GG), recessive model (TT vs TG and GG), and codominant model (TG vs GG), In addition, publication bias was detected by visual symmetry of funnel plots, with asymmetry suggesting possible publication bias. It was also assessed by Begg and Egger test in the meta-analysis. A *P*-value of less than .05 was considered to indicate publication bias. We also conducted a sensitivity analysis of the meta-analysis.

## 3 Results

### 3.1 Characteristics of the included studies

**Figure 1:**
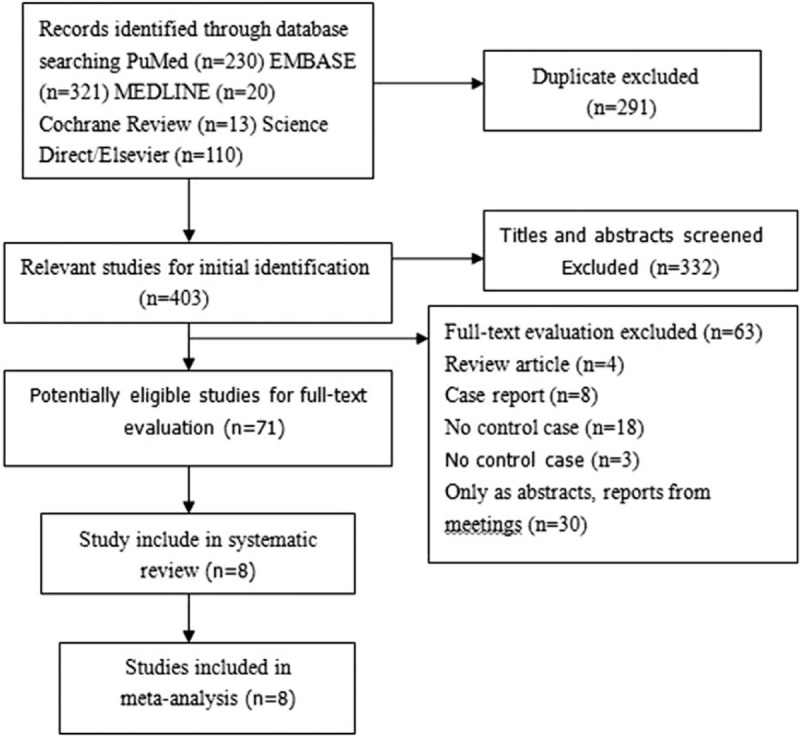
Flow diagram of the selection of eligible studies.

Figure 1 shows the details of the review process performed in this study. A total of 694 unduplicated studies were identified, 8 of which were ultimately selected in accordance with the eligibility criteria, and all reviewers were in agreement about the inclusion of all of these 8 papers. All these 8 studies were case-control study. Table 1 summarizes the data from the 8 studies. In total, the retrieved studies involved 7863 cases of PCa patients and 17,122 normal controls. All of these studies reported exclusion/inclusion criteria.^[15–22]^ In addition, all of these studies tested for the 17q24 rs1859962 polymorphism by using restriction fragment length polymorphism analysis after polymerase chain reaction amplification.

**Table 1:**
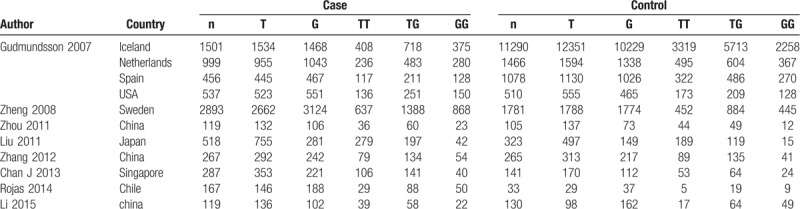
Characteristics of the included studies.

### 3.2 Meta-analysis

**Figure 2:**
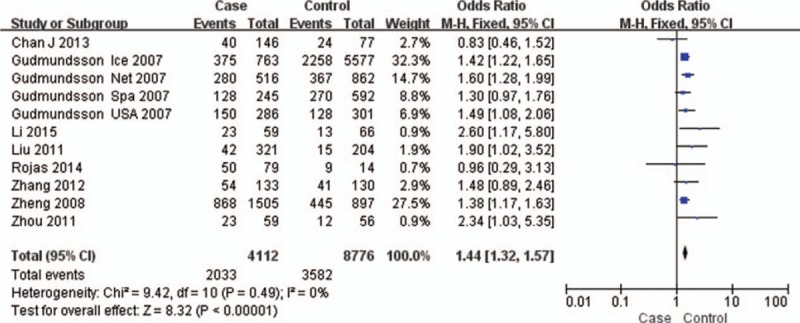
Forest plot showing the meta-analysis outcomes of the additive model.

**Figure 3:**
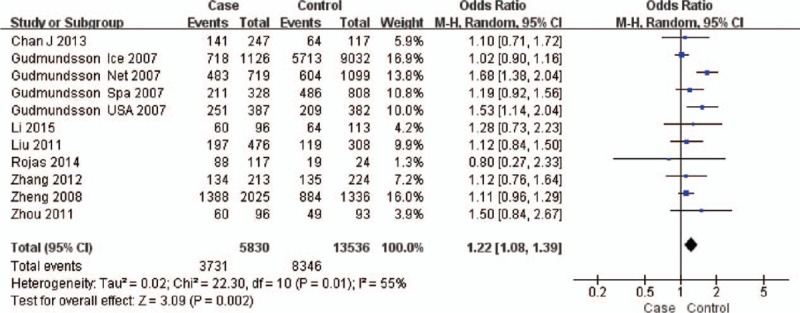
Forest plot showing the meta-analysis outcomes of the codominant model.

**Figure 4:**
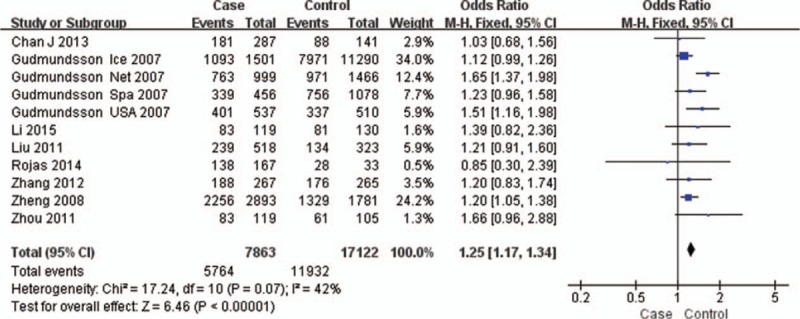
Forest plot showing the meta-analysis outcomes of the dominant model.

**Figure 5:**
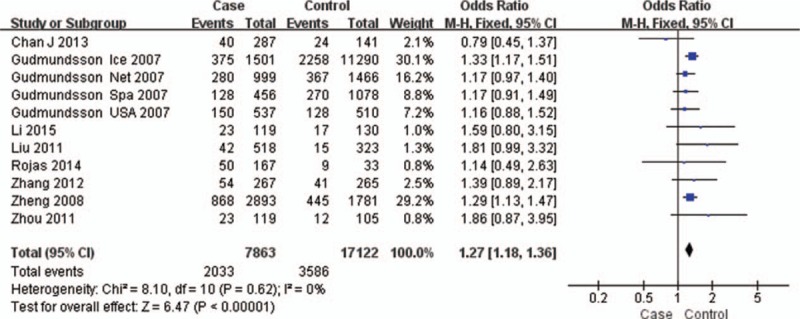
Forest plot showing the meta-analysis outcomes of the recessive model.

**Figure 6:**
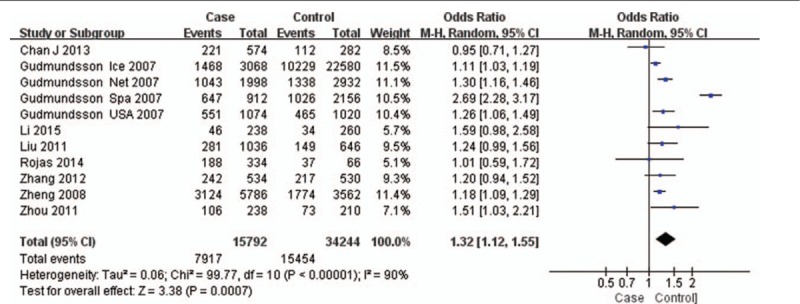
Forest plot showing the meta-analysis outcomes of the allele model.

**Figure 7:**
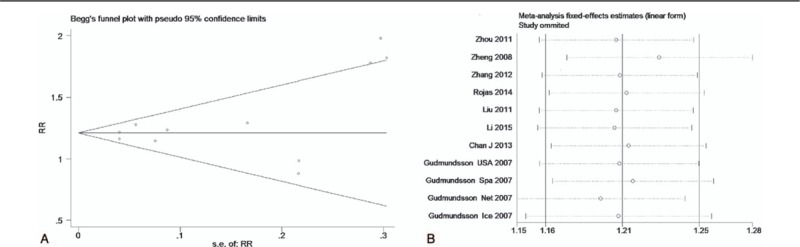
Begg publication bias and Sensitivity analysis plot of additive model, (A) Begg publication bias; (B) sensitivity analysis.

**Figure 8:**
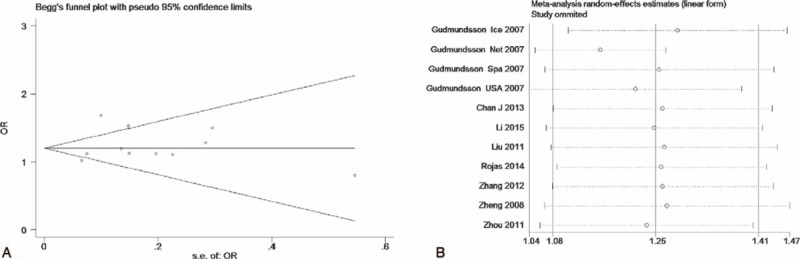
Begg publication bias and Sensitivity analysis plot of codominant model, (A) Begg publication bias; (B) sensitivity analysis.

**Figure 9:**
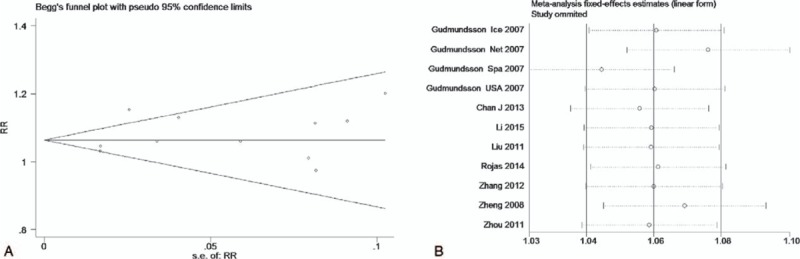
Begg publication bias and sensitivity analysis plot of dominant model, (A) Begg publication bias; (B) sensitivity analysis.

**Figure 10:**
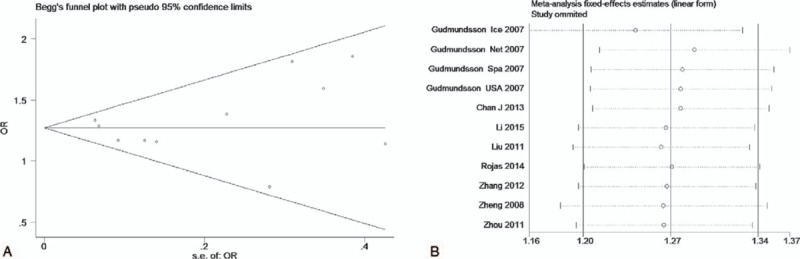
Begg publication bias and sensitivity analysis plot of recessive model, (A) Begg publication bias; (B) sensitivity analysis.

**Figure 11:**
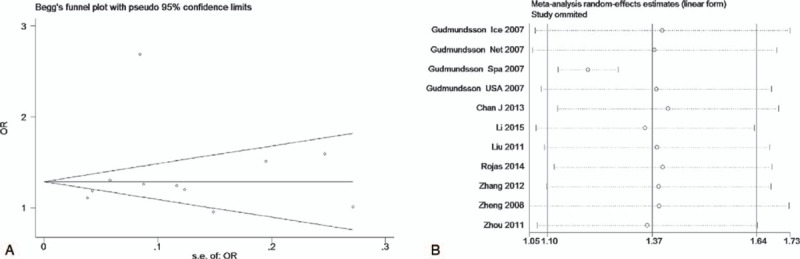
Begg publication bias and sensitivity analysis plot of allele model, (A) Begg publication bias; (B) sensitivity analysis.

The test of heterogeneity suggested that the data of the recessive model and additive model be analyzed in a fixed-effects model, and the dominant, codominant, and allele models be analyzed in a random-effects model. The meta-analysis revealed significant associations between the 17q24 rs1859962 gene polymorphism and PCa in all genetic models (*P* < .05). The combined ORs and 95% CIs were as follows: Additive model (ORs 1.44, 95%, CI [1.32, 1.57]) (Fig. 2); Codominant model (ORs 1.22, 95% CI [1.08, 1.39]) (Fig. 3); Dominant model (ORs 1.25, 95%, CI [1.17, 1.34]) (Fig. 4); Recessive model (ORs 1.27, 95% CI [1.18, 1.36]) (Fig. 5); Allele model (ORs 1.32, 95% CI [1.12, 1.55]) (Fig. 6). Begg funnel plots were largely symmetric (Figs. 7A, 8A, 9A, 10A, 11A), suggesting that there was no publication bias in the meta-analysis. Egger regression test also indicated little evidence of publication bias in all genetic models (*P* > .05) (Table 2). We also conducted a sensitivity analysis of the meta-analysis. We omitted 1 study at a time, and the calculated combined ORs for the remaining studies yielded consistent results. In the overall meta-analysis, no single study significantly changed the combined results, which indicated that the results were statistically stable and reliable (Figs. 7B, 8B, 9B, 10B, 11B).

**Table 2:**
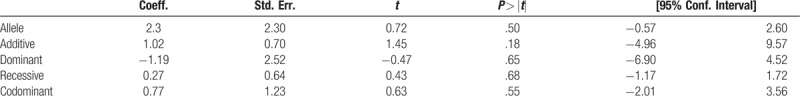
Egger test of publication bias.

## 4 Discussion

This study focused on 8 reports in the literature to clarify the association of the 17q24 rs1859962 gene polymorphism with the risk of PCa. For the additive model, dominant model, recessive model, codominant model, and allele model, 7, 3, 2, 2, and 6 studies reported a significant association between the 17q24 rs1859962 gene polymorphism and PCa, respectively, while the others did not. Our results reveal that, overall, significant associations between the 17q24 rs1859962 gene polymorphism and PCa were found in all genetic models. Specifically, the data indicate that 17q24 rs1859962 gene polymorphism might a risk factor of PCa.

17q24 SNPrs1859962 belongs to the noncoding pathogenic gene located in the long arm region 2 and 4 of chromosome 17, with a total length of about 600 kb Genes, its biological expression mechanism is not yet clear. SOX9 (SRY (sex determining region Y)-box 9), which is located in relatively close proximity (1 Mb) to the 17q24 rs1859962 risk variant. It is an important gene associated with early embryonic development, it does not directly regulate testicular development, but occurs through the combined action of the SRY gene on the male Y chromosome.

SOX9 belongs to the SOX (Sry-related high mobility group box) family of transcription factors and is a key regulator of developmental processes including male sex determination, chondrogenesis, neurogenesis, and neural crest development.^[23–26]^ Heterozygous SOX9 mutation is the cause of campomelic dysplasia, a severe form of human dwarfism characterized by extreme cartilage and bone malformation, which is frequently associated with XY sex reversal.^[27]^ The identified major targets of SOX9 are collagens (such as type II collagen [Col2a1] and type XI collagen [Col11a2]) during chondrogenesis and the Mullerian inhibiting substance during male sex differentiation.^[28]^ In adult tissues, SOX9 is expressed in intestinal crypts and hair follicles, where it is regulated by the Wnt/h-catenin or Sonic hedgehog signaling pathways and seems to be necessary to maintain stem cell/progenitor cell populations.^[29,30]^

Recently studies^[31]^ reported that SOX9 can interact with and regulate AR expression in PCa cells, which express high levels of AR in PCa cells indicates that SOX9-regulated genes may similarly play critical roles in supporting PCa growth.^[32]^ SOX9 also acts as a transcription factor in the development of prostate epithelia and its overexpression evidently plays a role in PCa tumorigenesis^[33,34]^ by the Wnt/h-catenin pathway, which has been implicated in the initiation and progression of many types of cancer. Manuel et al reported that SOX9 has also been identified as a downstream target of oncogene ERG^[35]^ and a recent large histopathological study found a strong correlation between positive ERG status and moderate and high levels of SOX9 in PCa tumor tissues.^[36]^

Our findings suggest that 17q24 rs1859962 gene polymorphism is a risk factor of PCa. The possible mechanisms may the target genes regulated by SOX9, and through stimulate androgen receptor, prostate-specific antigen expression, transcriptional regulation in PCa tumorigenesis, when over expressed.^[37]^ In this meta-analysis the G/G genotype is significantly associated with PCa risk. The transformation from codon T to G at this site, may influenced the biological function express of normal prostate cells, resulting in the increase risk of PCa. However, studies with larger sample sizes are needed to better illuminate the mechanisms of the 17q24 rs1859962 in the PCa tumorigenesis.

There are some limitations in our study, which need to be taken into consideration when interpreting the results of this meta-analysis. First, the sample size of each study was relatively small, and a total of 7863 PCa patients and 17122 normal controls were investigated in the 8 studies. Second, several studies on this issue were excluded owing to a lack of control data. Furthermore, because of the limited amount of original research, a subgroup of 17q24 rs1859962 gene polymorphism in different race was not conducted. As such, it is difficult to draw definitive conclusions about the clinical value of 17q24 rs1859962 gene variants in PCa.

In summary, the results of this meta-analysis suggests that 17q24 (rs1859962, G) is possibly a risk factor of PCa. The possible mechanism behind this may be as follows: the target genes regulated by SOX9, the transformation from codon T to G at this site, may influenced the biological function express of normal prostate cells, and it through stimulate androgen receptor, prostate-specific antigen expression, transcriptional regulation resulting in the increase risk of PCa. However, many important questions remain unanswered, including a more detailed analysis of the interactions between 17q sequence variants with PCa risk variants elsewhere in the genome and whether such modulation reflects SOX9 direct or indirect effects. In any case, SOX9-regulated genes may participate in important processes such as tumor angiogenesis, growth, or invasion. So studies with larger sample sizes are needed to shed more light on the correlation between 17q24 rs1859962 gene variants and PCa.

## Author contributions

**Conceptualization:** Feiqiang Ren, Peihai Zhang, Ziyang Ma, Ling Zhang, Guangsen Li, Xiaopeng Huang, Degui Chang, Xunjun Yu.

**Data curation:** Feiqiang Ren, Peihai Zhang, Ziyang Ma, Ling Zhang, Guangsen Li, Xiaopeng Huang, Degui Chang, Xunjun Yu.

**Formal analysis:** Feiqiang Ren, Peihai Zhang, Ziyang Ma, Ling Zhang, Guangsen Li, Xiaopeng Huang, Degui Chang, Xunjun Yu.

**Funding acquisition:** Feiqiang Ren, Peihai Zhang, Ziyang Ma, Ling Zhang, Guangsen Li, Xiaopeng Huang, Degui Chang, Xunjun Yu.

**Investigation:** Feiqiang Ren, Peihai Zhang, Ziyang Ma, Ling Zhang, Guangsen Li, Xiaopeng Huang, Degui Chang, Xunjun Yu.

**Methodology:** Feiqiang Ren, Peihai Zhang, Ziyang Ma, Ling Zhang, Guangsen Li, Xiaopeng Huang, Degui Chang, Xunjun Yu.

**Project administration:** Feiqiang Ren, Peihai Zhang, Ziyang Ma, Ling Zhang, Guangsen Li, Xiaopeng Huang, Degui Chang, Xunjun Yu.

**Resources:** Feiqiang Ren, Peihai Zhang, Ziyang Ma, Ling Zhang, Guangsen Li, Xiaopeng Huang, Degui Chang, Xunjun Yu.

**Software:** Feiqiang Ren, Peihai Zhang, Ziyang Ma, Ling Zhang, Guangsen Li, Xiaopeng Huang, Degui Chang, Xunjun Yu.

**Supervision:** Feiqiang Ren, Peihai Zhang, Ziyang Ma, Ling Zhang, Guangsen Li, Xiaopeng Huang, Degui Chang, Xunjun Yu.

**Validation:** Feiqiang Ren, Peihai Zhang, Ziyang Ma, Ling Zhang, Guangsen Li, Xiaopeng Huang, Degui Chang, Xunjun Yu.

**Visualization:** Feiqiang Ren, Peihai Zhang, Ziyang Ma, Ling Zhang, Guangsen Li, Xiaopeng Huang, Degui Chang, Xunjun Yu.

**Writing – original draft:** Feiqiang Ren, Peihai Zhang, Ziyang Ma, Ling Zhang, Guangsen Li, Xiaopeng Huang, Degui Chang, Xunjun Yu.

**Writing – review and editing:** Feiqiang Ren, Peihai Zhang, Ziyang Ma, Ling Zhang, Guangsen Li, Xiaopeng Huang, Degui Chang, Xunjun Yu.
